# Antitumor Effects of Cannabinoids in Human Pancreatic Ductal Adenocarcinoma Cell Line (Capan-2)-Derived Xenograft Mouse Model

**DOI:** 10.3389/fvets.2022.867575

**Published:** 2022-07-22

**Authors:** Siriwan Sakarin, Nuntana Meesiripan, Suleeporn Sangrajrang, Nuntakan Suwanpidokkul, Piyaporn Prayakprom, Chatchada Bodhibukkana, Vipada Khaowroongrueng, Kankanit Suriyachan, Somchai Thanasittichai, Attasit Srisubat, Pattamaporn Surawongsin, Kasem Rattanapinyopituk

**Affiliations:** ^1^Division of Research and Academic Support, National Cancer Institute, Bangkok, Thailand; ^2^The Government Pharmaceutical Organization, Bangkok, Thailand; ^3^Institute of Medical Research and Technology Assessment, Ministry of Public Health, Nonthaburi, Thailand; ^4^Division of Medical Technical and Academic Affairs, Ministry of Public Health, Nonthaburi, Thailand; ^5^Research and Technology Assessment Department, Ophthalmology Department, Lerdsin Hospital, Bangkok, Thailand; ^6^Center of Excellent for Companion Animal Cancer, Department of Veterinary Pathology, Faculty of Veterinary Science, Chulalongkorn University, Bangkok, Thailand

**Keywords:** cannabinoids, Capan-2, mouse, pancreatic cancer, xenograft tumor model

## Abstract

**Background:**

Pancreatic cancer is considered a rare type of cancer, but the mortality rate is high. Cannabinoids extracted from the cannabis plant have been interested as an alternative treatment in cancer patients. Only a few studies are available on the antitumor effects of cannabinoids in pancreatic cancer. Therefore, this study aims to evaluate the antitumor effects of cannabinoids in pancreatic cancer xenografted mouse model.

**Materials and Methods:**

Twenty-five nude mice were subcutaneously transplanted with a human pancreatic ductal adenocarcinoma cell line (Capan-2). All mice were randomly assigned into 5 groups including negative control (gavage with sesame oil), positive control (5 mg/kg 5-fluorouracil intraperitoneal administration), and cannabinoids groups that daily received THC:CBD, 1:6 at 1, 5, or 10 mg/kg body weight for 30 days, respectively. Xenograft tumors and internal organs were collected for histopathological examination and immunohistochemistry.

**Results:**

The average tumor volume was increased in all groups with no significant difference. The average apoptotic cells and caspase-3 positive cells were significantly increased in cannabinoid groups compared with the negative control group. The expression score of proliferating cell nuclear antigen in positive control and cannabinoids groups was decreased compared with the negative control group.

**Conclusions:**

Cannabinoids have an antitumor effect on the Capan-2-derived xenograft mouse model though induce apoptosis and inhibit proliferation of tumor cells in a dose-dependent manner.

## Introduction

Pancreatic cancer is considered a rare type of cancer. According to the hospital-based cancer registry, pancreatic cancer can be found in <1% of all cancer patients in Thailand similar to the United States, as reported by the American Cancer Society (1, 2). Despite the number of affected patients being low relative to other cancers, it is the fourth leading cause of cancer-related death in both males and females patients (2). Up to date, the major challenge in treatment is that this cancer is hard to be diagnosed and the affected patients will be diagnosed in the advanced stage. The operation in combination with radiation or chemotherapy is the standard treatment for pancreatic cancer (3). However, the mortality rate is still high and many patients are suffering from the side effects of treatment (4). In addition, pancreatic cancer is usually resistant to the most available chemotherapy drugs (5), making new drugs development more interested.

Nowadays, herbal medicinal plants such as cannabis are interested to use as an alternative treatment for patients with cancer. Cannabis was used in many cancer patients to cure or reduce symptoms caused by cancer or cancer treatments. It is believed that herbal medical plants are safer than conventional chemotherapy. The uses of cannabis have been reported to improve the quality of life in patients with cancer by reducing nausea and vomiting, appetite stimulating, and reducing pain (6). The antitumor effects of cannabis were demonstrated in several preclinical studies and investigated in various animal cancer models (7, 8). For instance, cannabinoids can reduce tumor progression by inducing apoptosis and inhibiting the proliferation of tumor cells (9). The role of cannabinoids as antitumor drugs has been reported in patients with pancreatic cancer due to cannabinoid receptors are expressed in pancreatic cells and upregulated in pancreatic cancer cells (5). Furthermore, cannabis extract has been reported to be used as a therapeutic agent in drug-resistance cancer (10). Many patients with pancreatic cancer, who are treated with advanced chemotherapy, are insufficient to improve their prognosis or survival due to the rapid development of drug resistance (11). Therefore, cannabis extract could be beneficially used as an alternative therapy in patients with pancreatic cancers.

The active components of cannabis called cannabinoids are composed of cannabidiol (CBD) and delta-9-tetrahydrocannabinol (THC). Although the antitumor effects of CBD have been widely focused on in oncology fields (12), the antitumor effect was increased when used CBD in combination with THC compared to a single compound use. Moreover, this combination showed better tolerated than the separate use (13). There are several studies that evaluated the antitumor effects of cannabinoids in animal models of cancers. However, there are few studies performed on animal models of pancreatic cancer. Therefore, this study aims to evaluate the antitumor effect of cannabinoids in a human pancreatic ductal adenocarcinoma cell line (Capan-2)-derived xenograft mouse model.

## Materials and Methods

### Cannabinoid Preparation

The THC:CBD (1:6) solution was prepared and obtained from the Government Pharmaceutical Organization, Thailand. *Cannabis sativa* L. strain was selected for this study and growing in GPO's greenhouse medical cannabis plantation. The extraction process followed the modified protocols described by previous studies (14). Briefly, the cola of cannabis was collected, and cannabinoids were extracted by using cold ethanol extraction. The solvent was subsequently evaporated using a rotary evaporator. After that cannabinoids extract was dissolved in pharmaceutical-grade oil.

### *In Vitro* Sensitivity Test of Cannabinoids

Briefly, a cell viability assay was used to determine cell survival following cannabinoids treatment. Cannabis extracts inhibited the proliferation of cultured cancer cells in a previous study. The findings revealed information on cell viability compared to normal cells with the cannabis extract concentration for inhibiting cell proliferation by 50% (half maximal inhibitory concentration or IC50). Cannabis extracts with a 1:1 THC:CBD ratio were found to be the most efficient in reducing the growth of cultured cancer cell lines, specifically Capan-2 (pancreatic cancer cells), followed by MCF-7 (breast cancer cells) and RBE (cholangiocarcinoma cells). The result of *in vitro* sensitivity test of cannabinoids for the Capan-2 cell line has been demonstrated recently, *in vitro* study of the Capan-2 xenografted animal model was performed in this study. The amount of cannabis extracts fed to nude mice was calculated. The concentration of cannabis extract that can inhibit cell proliferation of 50% in Capan-2 is 0.456 g/ml (unpublished data). These were used to compute the first dose that should be achieved using the pharmacokinetic technique of feeding. As a result, nude mice were given 1, 5, or 10 mg of cannabis extract per kilogram of body weight (BW).

### Cell Line and Culture Procedure

Human pancreatic adenocarcinoma cell line, Capan-2 (HTB-80™, ATCC®, Manassas, VA, USA), was cultured in cultured McCoy's 5a cell culture with L-glutamine sterile-filtered medium (ATCCTM 30-2007, Manassas, VA, USA) and added 10% fetal bovine serum (Cell-culture tested, ATCCTM 30-2020, Manassas, VA, USA) as protocol from the American Type Culture Collection (ATCC), in 25 cm^3^ sterile cell culture flasks (NUNC easy flask 25 filter, Thermo scientific, Shanghai, China). Capan-2 cells were incubated in a cell incubator containing 5% CO_2_ at 37°C and the culture medium was changed every 3 days. When the cell culture was 70–80% confluency, cell separation was carried out. The passage was carried out using 0.25% Trypsin solution-EDTA (Gibco, Thermo Scientific, Waltham, MA, USA) for cell digestion from the surface of sterile cell culture flasks. Cell lines were centrifuged to remove 0.25% Trypsin-EDTA. After that, the cell precipitation was dissolved with the media and placed in 75 cm^3^ sterile cell culture flasks (NUNC easy flask 75 filter, Thermo Scientific, Shanghai, China) and incubated in an incubator containing 5% CO_2_ at 37°C. The cell solution was then diluted with cell culture at a ratio of 1:10 for further passage into 75 cm^3^ sterile cell culture flasks.

Before transplantation, cell lines were digested from the 75 cm^3^ sterile cell culture flask with 0.25% Trypsin-EDTA solution, and then added to 10 ml working phosphate buffered saline pH 7.2 (Gibco, Thermo Scientific, Waltham, MA, USA). All amounts of cells were aspirated in a 15 ml centrifuge tube, centrifuged at 1,500 rpm for 5 min, then aspirated to remove the supernatant, repeated 2 times. After adding 0.1 ml McCoy's 5a with L-glutamines-filtered medium with 10% fetal bovine serum, the cell mixture was evaluated by the cell count by aspirating into the tube and adding 1 ml of 0.4% Trypan blue stain (Gibco, Thermo Scientific, Waltham, MA, USA) in the hemocytometer. The cells were then adjusted to 5 × 10^6^ cells in a 0.1 ml volume for injecting into the right flank of nude mice for tumor transplantation.

### Mouse Xenograft

The study protocol was approved by the Institutional Animal Care and Use Committee (IACUC) of The National Cancer Institute, Thailand (Protocol No.272_2019RB_IN602) and Lerdsin Hospital, Department of Medical Services (Protocol No. AEC-F-v03-02). Four-week-old male immunodeficient mice (nude mice, BALB/cAJcl-nu) were purchased from Nomura Siam International (Bangkok, Thailand) and maintained in a strictly hygienic conventional laboratory animal facility at Lerdsin Hospital under a 12:12 h dark/light cycle at a temperature of 22°C and humidity ranges of 50–70%. Twenty-five mice were acclimatized for 1 week before the experiment. Capan-2 cells (5 × 10^6^ cells) were subcutaneously injected at the flank of mice under aseptic conditions. The weight of mice and tumor size were determined every 3 days. Tumor size was measured by caliper and tumor volume was calculated by the following equation: Tumor volume (mm^3^) = 1/2 (length × width^2^) as described by Song et al. (15). When tumor volume reached 200 mm^3^, all mice were randomly assigned into 5 groups, 5 mice each, including the negative control group (group 1), the positive control group (group 2), and experimental groups (groups 3–5). Mice were gavaged with sesame oil in the negative control group, intraperitoneally injected with 5-fluorouracil (5-FU) at a dose of 5 mg/kg BW for 3 times a week in the positive control group, and daily gavaged with 1, 5, or 10 mg/kg BW cannabinoids (THC:CBD, 1:6) as the low-, intermediate-, and high-dose groups, respectively, for 30 days. THC:CBD (1:6) solution was prepared and obtained from the Government Pharmaceutical Organization, Thailand.

All mice were sacrificed on day 30 after the first dose of cannabis was given, 1 ml of blood sample was collected for hematological and blood chemistry profiles. Xenograft tumors and internal organs were collected and fixed in 10% buffered formalin solution for 24 h and embedded in paraffin blocks. Tissue sections were cut and stained with H&E for evaluating general histopathological appearance, identifying the metastasis of tumor cells in other organs, and determining the necrotic area and the apoptotic cell in tumor sections under the light microscope. The percentage of the necrotic area (%necrotic area) was measured by the image analyzer program (NIS-Elements Analysis D) (Nikon, Tokyo, Japan). The calculation of the percentage of the necrotic area was by dividing the necrotic area by the total tumor area. Moreover, the tumor sections were randomly selected at a high-power field (40× magnification) for determining the average number of apoptotic cells. Apoptotic cells were manually counted from 5 to 8 high-power fields of each mouse and calculated as the average number of apoptotic cells per high-power field.

The proliferation and apoptosis of tumor cells were evaluated by immunohistochemistry technique using monoclonal mouse anti-proliferating cell nuclear antigen (PCNA) antibody (clone PC10, Dako, Hamburg, Germany) at dilution 1:400 and rabbit polyclonal anti-caspase-3 (ab4051, Abcam, Cambridge, UK) at dilution 1:200, respectively. Five to seven high-power fields of each mouse were randomly selected for evaluation. The immunoreactivity of PCNA was expressed as intensity score as follows: score 0, no staining; score 1, slight staining or 0–25% of total cells; score 2, 26–50% of total cells; score 3, 51–75% of total cells; and score 4, >75% of total cells (16). Caspase-3 positive cells were manually counted in high power fields and calculated as the average positive cells per area.

### Statistical Analysis

Statistical analysis was performed by computer-based software (SPSS, IBM, Chicago, IL, USA). The normality test was evaluated before statistical analysis and normality data were expressed as mean ± SD. The differences among groups were tested with one-way ANOVA and Tukey test was used for *post-hoc* analysis. *P*-value < 0.05 is considered statistically significant.

## Results

### Weight and Tumor Volume

**Figure 1:**
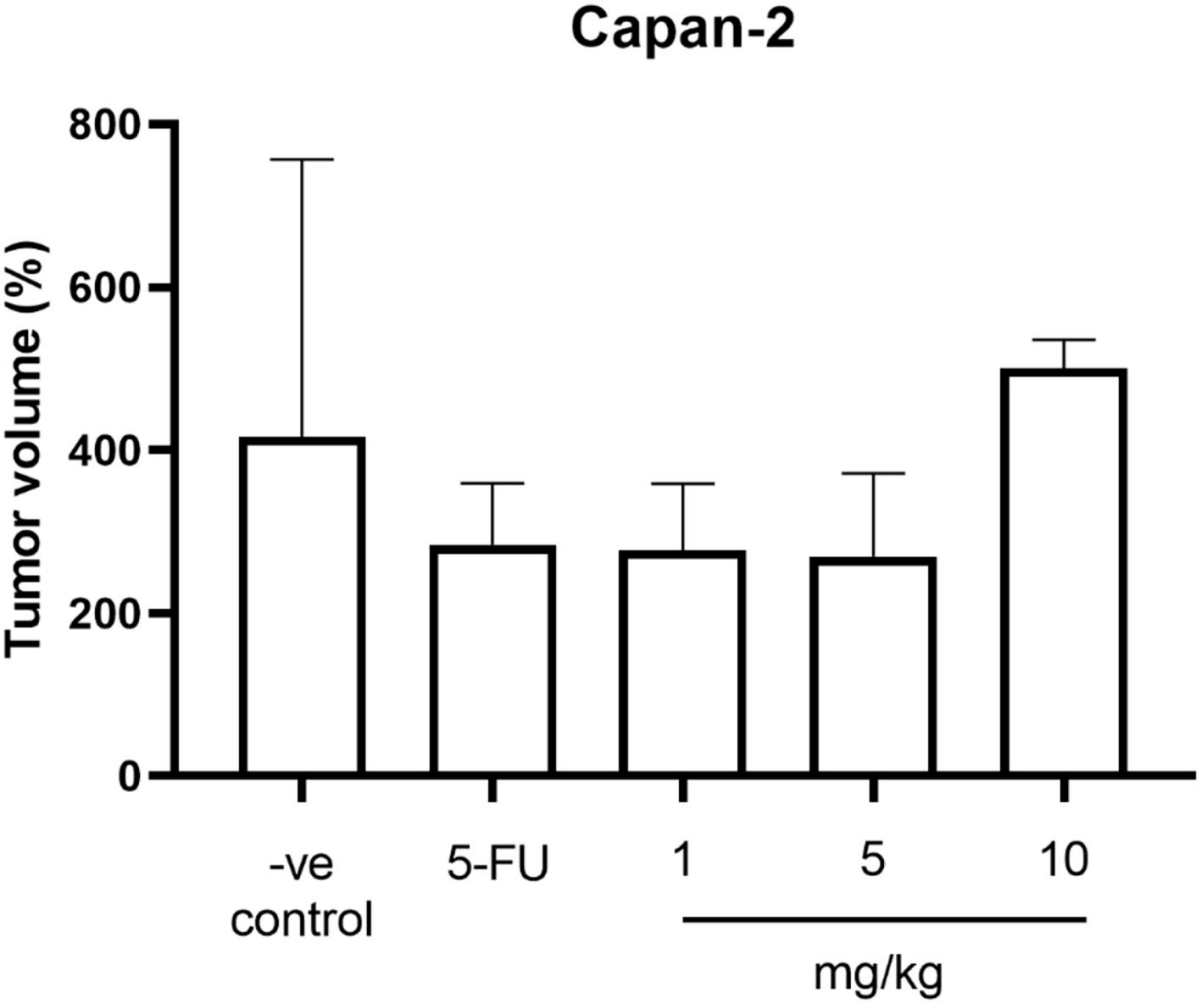
The percentage of tumor volume changes of mice in the negative control group, the positive control group (5-FU), and THC:CBD at dose of 1, 5, and 10 mg/kg BW.

The average weight of mice in all groups before the experiment was 25.09 ± 0.98 g. The average weight on day 30 at the end of the experiment was 26.16 g and the percentage of weight changes between groups was not significantly different (*P* > 0.05). However, the percentage of weight changes was increased in all groups. The average tumor volume before the experiment and on day 30 between groups was not significantly different (*P* > 0.05). The percentage of tumor volume changes was increased in all groups. When compared to the negative control group, the percentage of tumor volume in the positive control group (283.96 ± 37.52%) and treatment groups including 1 and 5 mg/kg (276.93 ± 36.41% and 269.32 ± 51.08%, respectively) was lower while the treatment group at 10 mg/kg (499.94 ± 20.51%) was higher than the negative control group (416.15 ± 152.36%) but all these changes do not reach the significant level (*P* > 0.05) (Table 1; Figure 1).

**Table 1:** The average tumor volume before the experiment (day 0) and on day 30 and the percentage of tumor volume changes.

**Group**	**Day 0** **(mm^**3**^)**	**Day 30** **(mm^**3**^)**	**Percentage of tumor volume change (%)**
1. Negative control	240.49 ± 64.92	982.26 ± 200.22	416.15 ± 152.36
2. Positive control (5-FU)	189.44 ± 13.99	726.01 ± 91.47	283.96 ± 37.52
3. Low-dose THC:CBD (1 mg/kg BW)	252.82 ± 14.03	933.07 ± 41.52	276.93 ± 36.41
4. Intermediate-dose THC:CBD (5 mg/kg BW)	200.58 ± 23.95	773.73 ± 207.89	269.32 ± 51.08
5. High-dose THC:CBD (10 mg/kg BW)	110.40 ± 38.07	650.36 ± 203.82	499.94 ± 20.51

*Data were expressed as mean ± SD*.

### Complete Blood Count and Blood Chemistry Profiles

There was no significant difference in blood profiles between groups except for total numbers of white blood cells that showed higher in mice treated with THC:CBD at doses 1 and 5 mg/kg BW compared with mice treated with 5-FU (*P* < 0.05). However, the values were within the normal limit, suggesting no clinical significance (Table 2).

**Table 2:** Hematological and blood chemistry profiles of mice.

**Parameter**	**Unit**	**Group**				
		**Negative control**	**Positive control** **(5-FU)**	**Low-dose** **THC:CBD** **(1 mg/kg BW)**	**Intermediate-dose THC:CBD** **(5 mg/kg BW)**	**Low-dose** **THC:CBD** **(10 mg/kg BW)**
RBC count	x10^6^ cell	10.1 ± 0.07	9.4 ± 0.24	10.0 ± 0.30	10.2 ± 0.31	9.5 ± 0.79
Hct	%	44.0 ± 0.00	44.2 ± 0.84	46.3 ± 0.58	46.3 ± 1.89	43.2 ± 3.27
Hb	g/dl	15.2 ± 0.14	14.9 ± 0.49	15.2 ± 0.50	15.4 ± 0.67	14.5 ± 0.97
MCV	fl	44.1 ± 0.30	46.8 ± 0.70	45.9 ± 1.00	45.4 ± 0.80	45.5 ± 0.80
MCH	pg	15.1 ± 0.07	15.8 ± 0.23	15.1 ± 0.56	15.1 ± 0.26	15.2 ± 0.35
MCHC	g/dl	34.2 ± 0.42	33.7 ± 0.68	33.0 ± 0.75	33.3 ± 0.57	33.5 ± 0.86
RDW	%	19.0 ± 0.57	21.7 ± 4.46	18.6 ± 0.30	18.8 ± 0.31	18.4 ± 1.12
Platelet	x10^6^ cell	0.74 ± 0.833	1.37 ± 0.122	1.10 ± 0.50	1.12 ± 0.303	1.15 ± 0.400
WBC count	cell	6,450 ± 3,889	5,100 ± 1,235	10,333 ± 2,760^a^	9,375 ± 2,155^a^	5,740 ± 789
Neutrophil	%	60.0 ± 9.90	58.0 ± 6.04	46.0 ± 14.18	47.5 ± 7.19	61.8 ± 6.18
Lymphocyte	%	38.5 ± 9.19	39.8 ± 5.22	51.0 ± 16.64	50.8 ± 8.02	35.2 ± 6.61
Monocyte	%	1.0 ± 0.00	1.4 ± 0.55	1.3 ± 0.58	1.0 ± 0.00	1.6 ± 0.89
Eosinophil	%	0.5 ± 1.00	0.8 ± 1.00	1.7 ± 2.00	0.8 ± 1.00	1.4 ± 2.00
Basophil	%	0.0 ± 0.00	0.0 ± 0.00	0.0 ± 0.00	0.0 ± 0.00	0.0 ± 0.00
ALT	U/l	68.7 ± 10.60	42.8 ± 7.56	63.5 ± 14.39	53.3 ± 8.18	59.6 ± 27.62
AST	U/l	238.7 ± 61.52	157.6 ± 9.36	230.8 ± 74.30	202.0 ± 16.04	223.4 ± 85.38
BUN	mg/dl	24.7 ± 2.31	22.0 ± 1.41	28.5 ± 13.82	21.0 ± 2.16	23.8 ± 3.35
Creatinine	mg/dl	0.5 ± 0.06	0.4 ± 0.05	0.6 ± 0.19	0.5 ± 0.05	0.5 ± 0.11
Albumin	g/dl	3.5 ± 0.46	2.4 ± 0.46	3.3 ± 0.81	2.8 ± 0.21	3.3 ± 1.59
Total protein	g/dl	8.1 ± 0.68	6.3 ± 0.62	8.3 ± 2.09	7.2 ± 0.37	8.0 ± 2.33

*Data were expressed as mean ± SD*.

a *It indicates a significant difference at P < 0.05 compared to the positive control group (5-FU)*.

*RBC, red blood cell; Hct, hematocrit; Hb, hemoglobin; MCV, mean corpuscular volume; MCH, mean corpuscular hemoglobin; MCHC, mean corpuscular hemoglobin concentration; RDW, red blood cell distribution; ALT, alanine aminotransferase; AST, aspartate aminotransferase; BUN, blood urea nitrogen*.

### Gross and Histological Morphology

**Figure 2:**
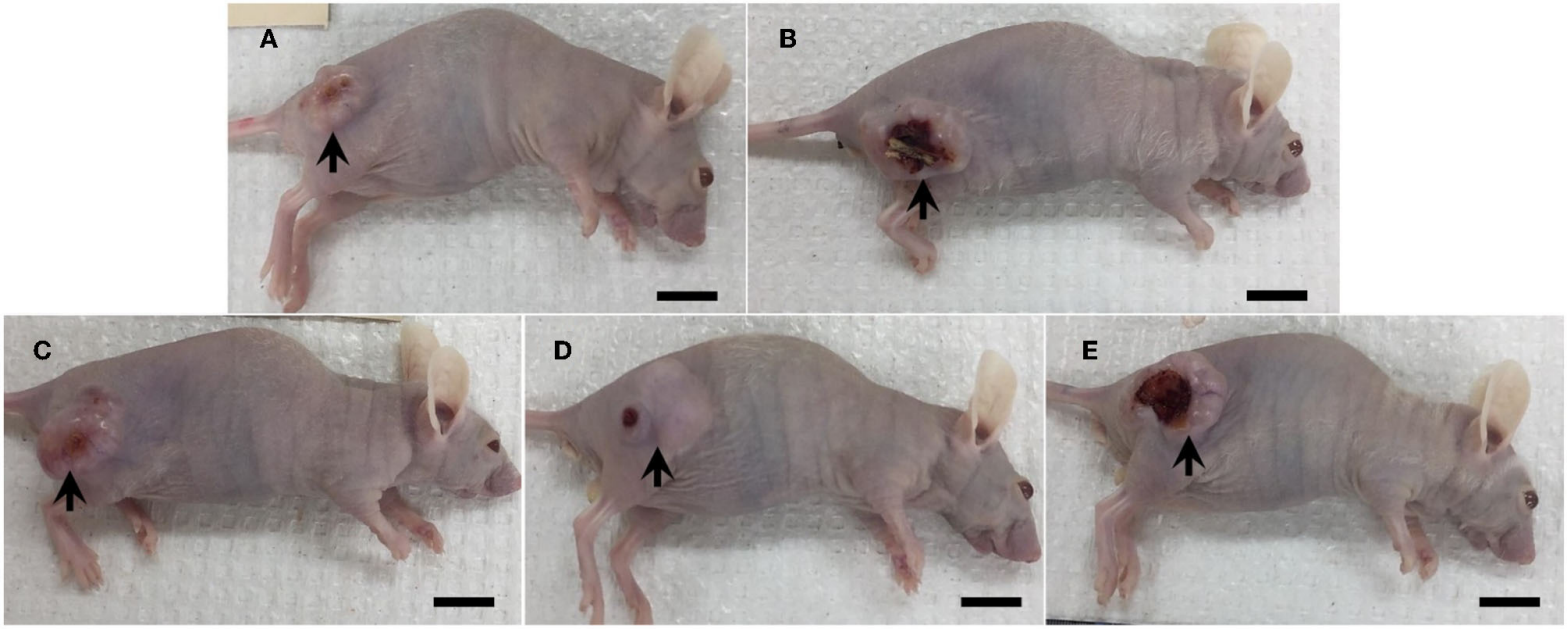
Gross morphology of mice in the negative control group **(A)**, the positive control group (5-FU) **(B)**, and THC:CBD at the dose of 1 mg/kg BW **(C)**, 5 mg/kg BW **(D)**, and 10 mg/kg BW **(E)**, (scale bar = 1 cm). Tumor mass was presented subcutaneously with ulcerated and necrosis in several mice (arrow).

**Figure 3:**
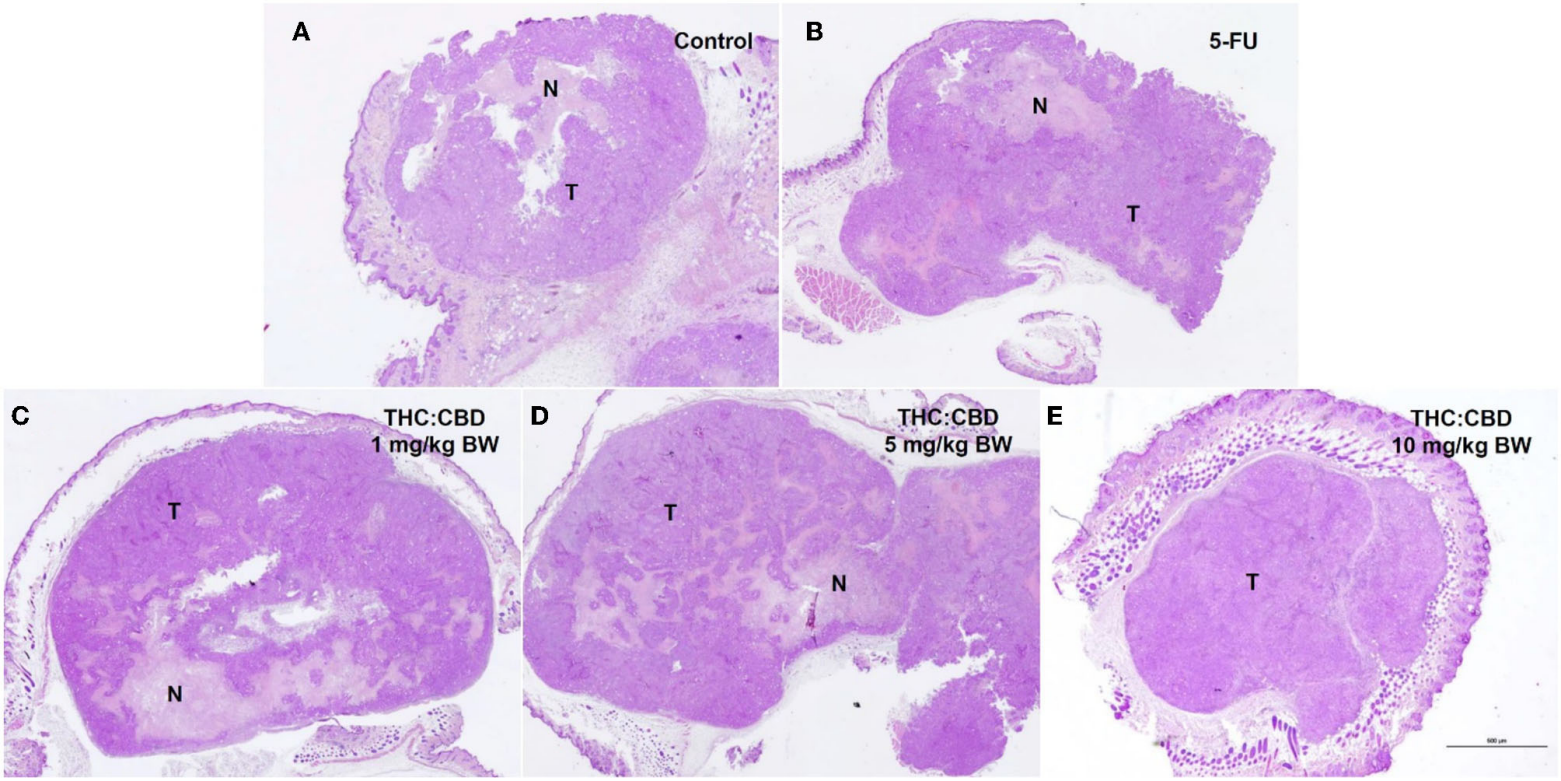
Histopathological morphology of mice in the negative control group **(A)**, the positive control group (5-FU) **(B)**, and THC:CBD at dose of 1 mg/kg BW **(C)**, 5 mg/kg BW **(D)**, and 10 mg/kg BW **(E)**, (HE, 2×). Tumor masses were presented with necrotic tissue (N). Necrotic tissues were presented in the center and at the edge of the tumor especially in treatment and positive control groups compared with the negative control group.

**Figure 4:**
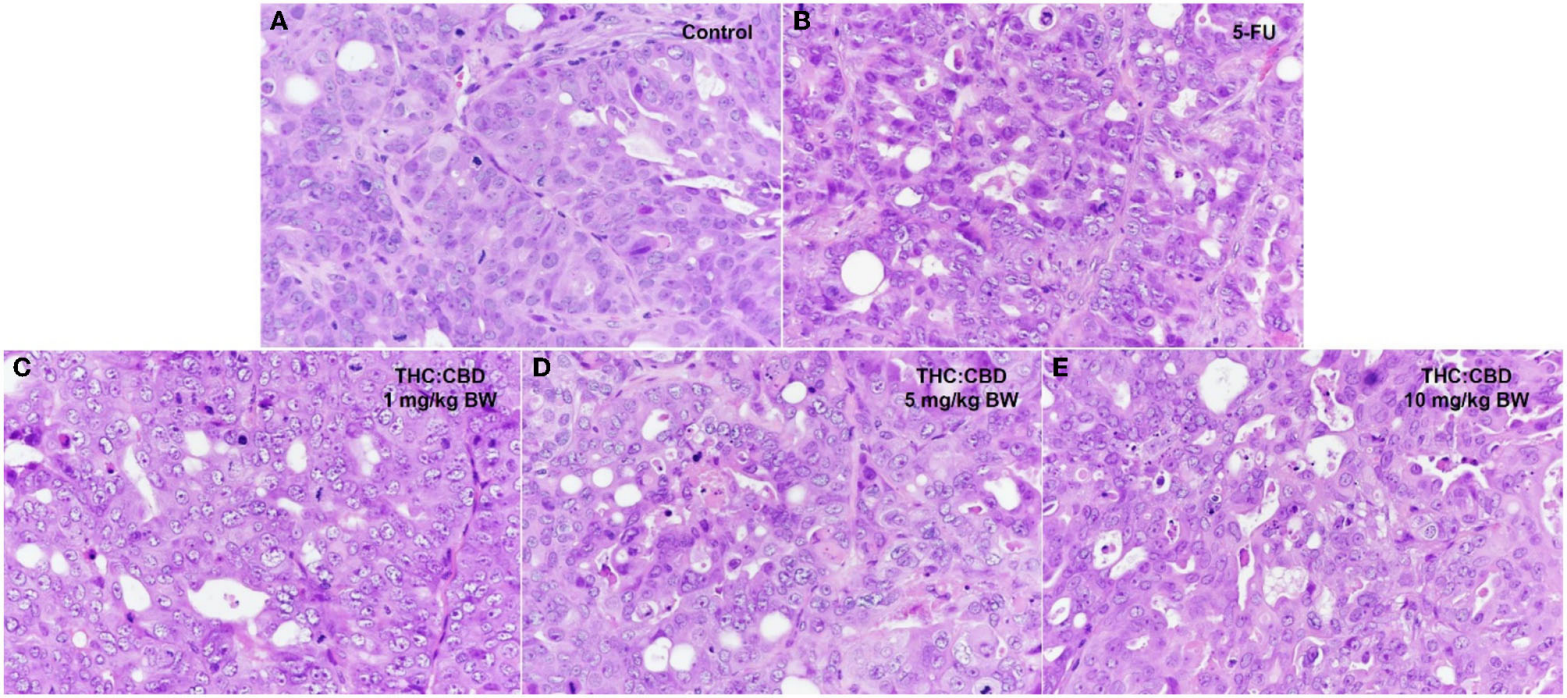
Histopathological morphology of mice in the negative control group **(A)**, the positive control group (5-FU) **(B)**, and THC:CBD at dose of 1 mg/kg BW **(C)**, 5 mg/kg **(D)**, and 10 mg/kg **(E)**, (HE, 40×).

**Figure 5:**
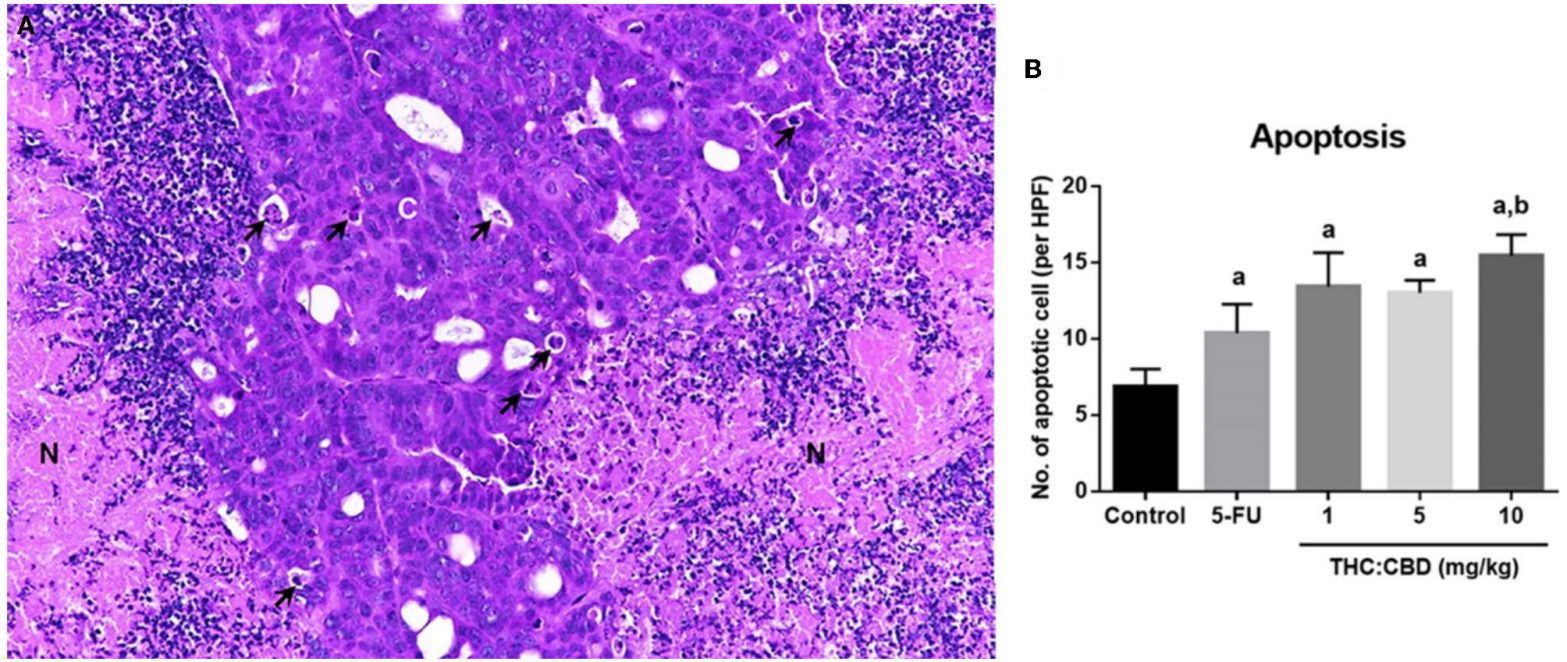
Histopathological morphology of pancreatic adenocarcinoma of mice gavaged with THC:CBD. The tumor demonstrated scattered apoptotic cells (arrow) with necrotic tissue (N) **(A)** (HE, 20×). The graph demonstrated the average apoptotic cells per high-power field in the negative control group, the positive control group (5-FU), and THC:CBD at the dose of 1, 5, and 10 mg/kg BW. Data were expressed as mean ± SD. a indicates a significant difference at *P* < 0.05 compared to the negative control group. b indicates a significant difference at *P* < 0.05 compared to the positive control group (5-FU) **(B)**.

Tumor masses were presented subcutaneously at the transplanted areas (the right flank) with some ulcerated and necrotic lesions in several mice of every group (Figure 2). The metastasis of tumors in other internal organs (both intrathoracic and intra-abdominal organs) was not grossly observed in every mouse. Microscopically, the morphology of tumors was consistent with pancreatic ductal adenocarcinoma in every sample (Figure 3). The histological findings revealed the various arrangement patterns of the tumor, namely, tubular, tubulopapillary, lobular, and solid patterns (Figure 4). Necrotic tissues were presented in the center and at the edge of the tumor especially in the treatment and positive control groups compared with the negative control group (Figure 3). Apoptotic cells were scattered throughout the tumor (Figure 5).

The percentage of necrotic area in the positive control group (32.88 ± 2.940%) and the treatment groups (40.64 ± 3.689, 34.88 ± 6.771, and 27.31 ± 8.841 for 1, 5, and 10 mg/kg BW THC:CBD, respectively) was higher than in the negative control group (24.79 ± 4.092) but does not reach a significant level (*P* > 0.05) (Table 3). The average apoptotic cells were the lowest in the negative control groups (6.87 ± 0.518). The higher numbers were shown in the positive control group and the treatment groups compared to the negative control group. The highest numbers were observed in the 10 mg/kg BW THC:CBD given group (15.47 ± 0.693) compared to the negative control, the positive control (10.40 ± 0.840 cells), and 1 and 5 mg/kg BW THC:CBD treated groups (13.45 ± 1.108 and 13.03 ± 0.408, respectively) (*P* < 0.05) (Table 3).

**Table 3:** The percentage of necrotic area and the average apoptotic cells.

**Group**	**Percentage of necrotic area (%)**	**Average apoptotic cells/HPF**
1. Negative control 2. Positive control (5-FU) 3. Low-dose THC:CBD (1 mg/kg BW) 4. Intermediate-dose THC:CBD (5 mg/kg BW)5. High-dose THC:CBD (10 mg/kg BW)	24.79 ± 4.092 32.88 ± 2.940 40.64 ± 3.689 34.88 ± 6.771 27.31 ± 8.841	6.87 ± 0.518 10.40 ± 0.840^a^ 13.45 ± 1.108^a^ 13.03 ± 0.408^a^ 15.47 ± 0.693^a,b^

*Data were expressed as mean ± SD*.

a *It indicates a significant difference at P < 0.05 compared to the negative control group*.

b *It indicates a significant difference at P < 0.05 compared to the positive control group (5-FU)*.

### Proliferation and Apoptosis of Tumor Cells

**Figure 6:**
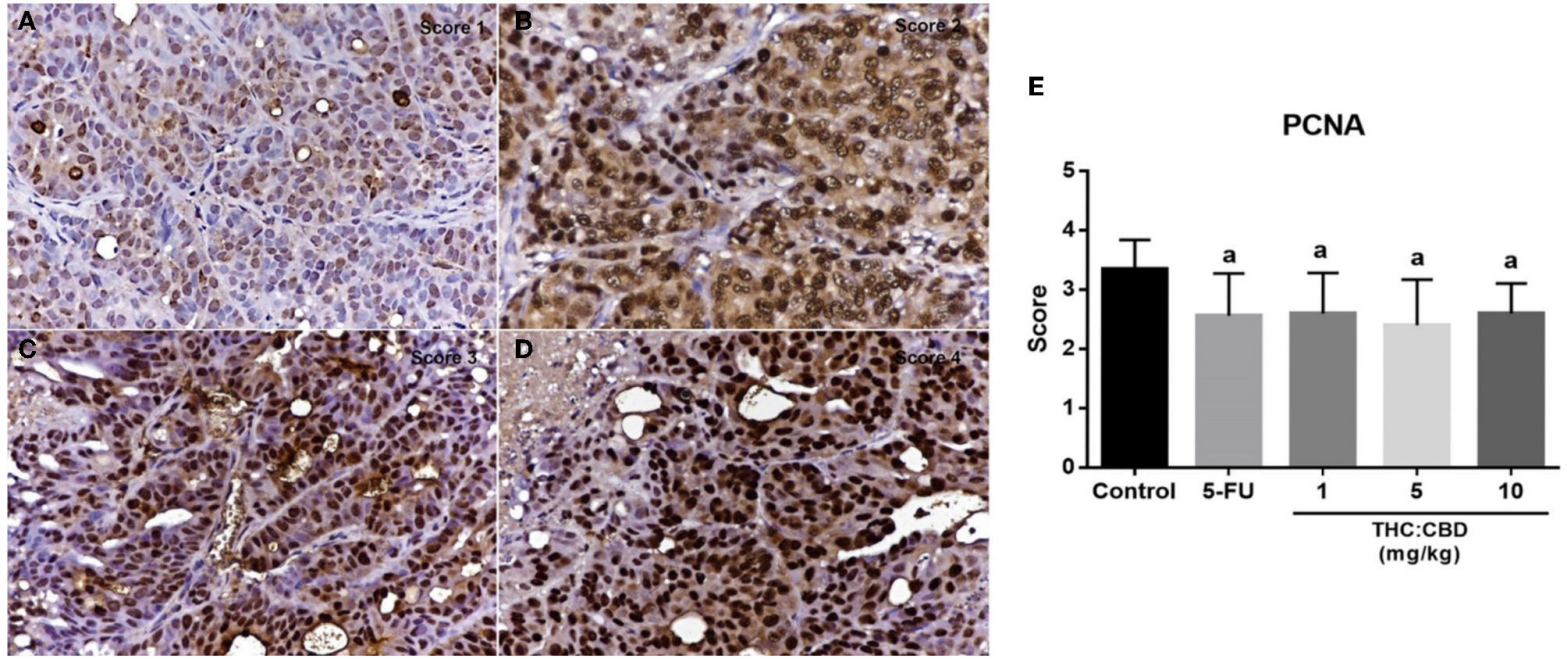
The expression score of PCNA in tumor **(A)** score = 1, **(B)** score = 2, **(C)** score = 3, and **(D)** score = 4 (counterstained with Mayer's hematoxylin, 40×). The graph demonstrates the expression score of PCNA in the negative control group, the positive control group (5-FU), and THC:CBD at the dose of 1, 5, and 10 mg/kg BW. Data were expressed as mean ± SD. a indicates a significant difference at *P* < 0.05 compared to the negative control group **(E)**.

**Figure 7:**
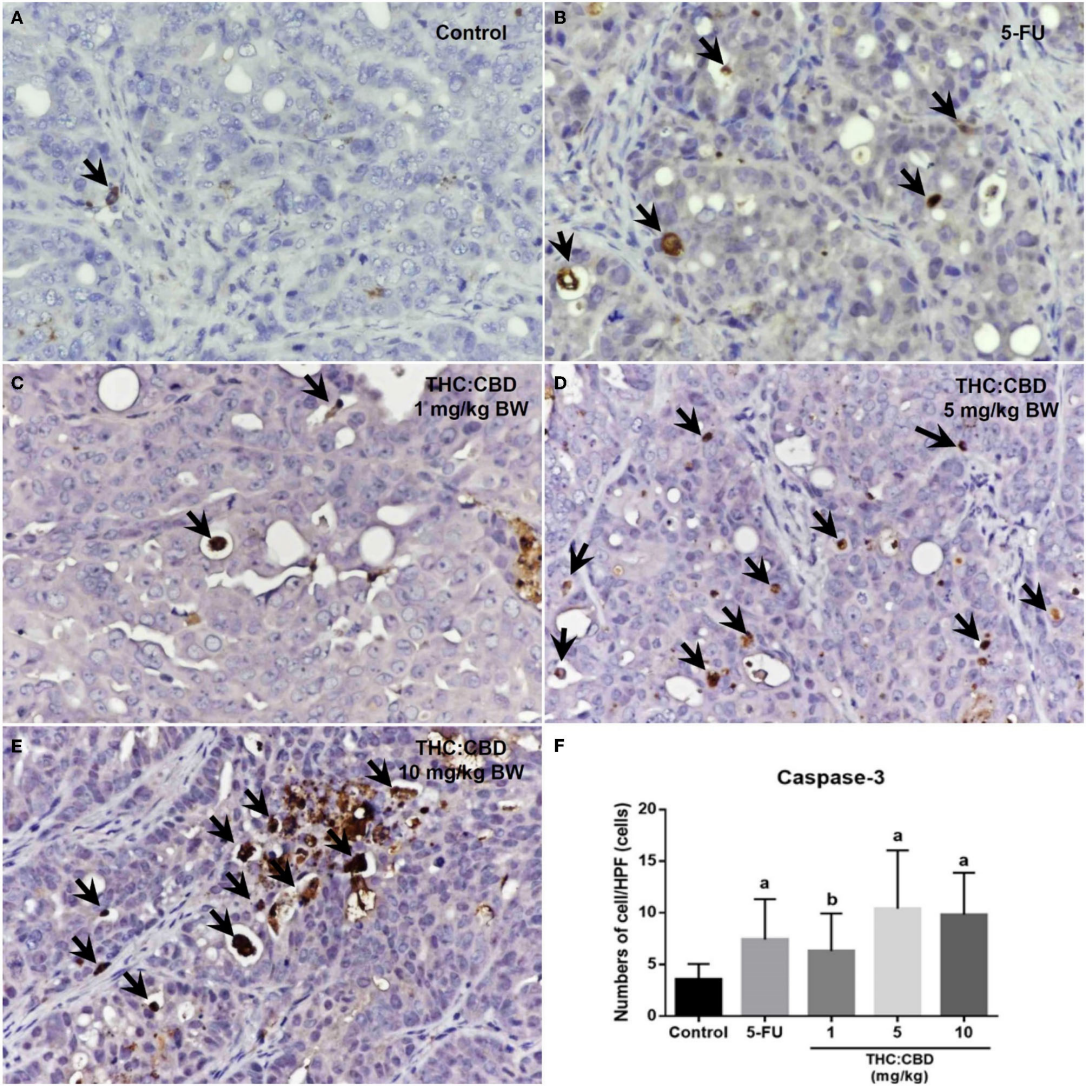
Caspase-3 expression in the negative control group **(A)**, the positive control group (5-FU) **(B)**, and THC:CBD at the dose of 1, 5, and 10 mg/kg BW **(C–E)**, respectively. Caspase-3 positivity is presented by brown color (arrows) (counterstained with Mayer's hematoxylin, 40×). The graph demonstrates the average number of caspase-3 positive cells per high-power field in the negative control group, the positive control group (5-FU), and THC:CBD doses of 1, 5, and 10 mg/kg BW. Data were expressed as mean ± SD. a indicates a significant difference at *P* < 0.05 compared to the negative control group. b indicates a significant difference at *P* < 0.05 compared to the positive control group (5-FU) **(F)**.

As previously mentioned, PCNA was used for evaluating the proliferation capacity of the tumor cells. The PCNA-positive cells were detected in tumors of all groups. The results showed that the PCNA expression score was expressed from scores 1–4 (Figure 6). The expression score in the positive control (2.56 ± 0.712) and the treatment groups (2.60 ± 0.681, 2.40 ± 0.770, and 2.60 ± 0.507 for 1, 5, and 10 mg/kg BW THC:CBD, respectively) was significantly lower than in the negative control group (3.35 ± 0.489) (*P* < 0.05) (Table 4). Caspase-3 expression was used as a biomarker of apoptotic cells and it was expressed in both nucleus and cytoplasm of apoptotic cells (Figure 7). In a similar way to PCNA expression, the average caspase-3 positive cells in the positive control group (7.43 ± 3.897) and THC:CBD at doses of 5 and 10 mg/kg BW (10.40 ± 5.651 and 9.80 ± 4.074, respectively) were increased compared with the negative control group (3.56 ± 1.502) (Table 4). Although, caspase-3 positive cells showed in 1 mg/kg BW THC:CBD (6.31 ± 3.614) were greater than the negative control group, however, it does not reach the significant level (*P* > 0.05).

**Table 4:** The expression score of PCNA and the average caspase-3 positive cells.

**Group**	**Average IHC score of PCNA**	**Caspase-3 positive cells/HPF**
1. Negative control 2. Positive control (5-FU) 3. Low-dose THC:CBD (1 mg/kg BW) 4. Intermediate-dose THC:CBD (5 mg/kg BW) 5. High-dose THC:CBD (10 mg/kg BW)	3.35 ± 0.489 2.56 ± 0.712^a^ 2.60 ± 0.681^a^ 2.40 ± 0.770^a^ 2.60 ± 0.507^a^	3.56 ± 1.502 7.43 ± 3.897^a^ 6.31 ± 3.614^b^ 10.40 ± 5.651^a^ 9.80 ± 4.074^a^

*Data were expressed as mean ± SD*.

a *It indicates a significant difference at P < 0.05 compared to the negative control group*.

b *It indicates a significant difference at P < 0.05 compared to the positive control group (5-FU)*.

## Discussion

The antitumor effects of cannabinoids such as inducing apoptosis, inhibiting proliferation, migration, and angiogenesis of tumor cells have been reported in several *in vivo* and *in vitro* studies (7–9, 17). In animal xenograft tumor models, cannabinoids decreased the tumor progression by inducing apoptosis and inhibiting the proliferation of tumor cells (7–9, 12, 18). The active components of cannabis are called cannabinoids composed of CBD and THC. In addition, the antitumor effect was increased when used CBD in combination with THC compared to single use of CBD or THC alone and their combination showed better tolerance than separate use (13).

Tumor cells xenografts transplanted subcutaneously into the immunodeficiency mice have been widely tested in the preclinical studies for identifying or developing the new anticancer drugs (19). The immunodeficient mice such as BALB/c nude mice were the mice that lack T-cell lymphocytes, allowing tumor cell lines to be proliferated and propagated into a solid mass in the subcutaneous region (20, 21). The current experiment selected the xenograft tumor model to evaluate the effects of cannabinoid compounds in pancreatic adenocarcinoma transplanted mice. 5-FU is a chemotherapeutic agent that inhibits the enzyme thymidylate synthase in thymidine formation, which is essential for DNA synthesis (22). 5-FU is one of the drugs used for the treatment of pancreatic cancer (23–27). Moreover, 5-FU is also used as a positive drug treatment group in many mouse models (28–30).

The effect of cannabinoid treatment on the weight gaining of mice was contradictory. The studies in mice that received cannabinoids by peritoneal injection showed no effect on food intake (31, 32) as same as the study in rats (33). On contrary, the studies in mice and rats that received cannabinoids by peritoneal injection or oral gavage showed a decrease in weight gain due to a decrease in food intake (34–37). However, this current study showed that the weight of mice in all groups was increased and the weight of mice in treatment groups receiving THC:CBD was not significantly different compared with the negative control group. This result suggested that cannabinoids may not affect the weight gain of mice in the present study.

The measurement of tumor size is important for assessing the responses to cancer treatments. The standard method for determining tumor size of subcutaneously xenografted tumors is manually measured by the caliper and calculated as tumor volume by using the following equation: 1/2 × (Length × Width^2^) (38). Although this method is easy to perform, it is subjective, and the error can occur due to different observers. Therefore, tumor size measurement was performed by the same person throughout the experiment. Tumor volume of mice was increased in all groups with no significant difference between groups. Grossly, necrotic tissues were presented in the center and at the edge of the tumor in all groups including the negative control group. It may explain that the necrosis of the tumor probably occurred from hypoxia and nutrient deprivation of rapid growth tumor (39) more than the effect of treatments. Although tumor volumes were increased in all groups, histopathological examination showed that treatment groups with high doses presented with a higher number of apoptotic cells compared with the negative control group and the expression of caspase-3. These results showed similarities with the previous studies in cell culture and animal models. The cannabinoids were found to induce pancreatic cancer cell lines (PANC-1 and MiaPaCa-2) apoptosis without affecting normal pancreatic cells (40). In animal models, cannabinoids with high concentrations can induce apoptosis of cholangiocarcinoma cells (41). Surprisingly, the high-dose treatment group presented with a higher number of apoptotic cells while the percentage of tumor volume tended to be higher than in other groups. Tissue remodeling with fibrosis after apoptosis has been reported in several tissues (42, 43). In this present study, the increase of fibrotic tissue in the tumor mass of the high-dose group has been found. Therefore, it was anticipated that the increase in tumor volume in the high-dose treatment group may result from an increase in collagen deposition in tumor mass. As for the tumor proliferative capacity, the expression score in the positive control and the treatment groups was lower than in the negative control group suggesting the proliferation of tumor cells decreased in the positive control and the treatment groups. Similar to the previous study in animal models that combination of cannabinoids and other drugs can prolong survival and inhibit tumor cell proliferation (44). The results from this study revealed that the treatment groups presented with an increase in apoptosis and a decrease in proliferation compared with the negative control whereas tumor volume in the treatment groups was not changed or reduced compared with the negative control group. It became evident that tumor volume was not associated with the apoptotic and proliferative indices of tumor cells. Such finding offers support to the previous study undertaken by Mattern and Volm (45).

Several studies reported chemoresistance in pancreatic cancer including 5-FU chemotherapy (25, 27, 46, 47). However, the previous studies reported that the use of cannabinoids in combination with chemotherapy showed synergistic effects and cannabinoids attenuated side effects of chemotherapy (48, 49) and increase survival in transgenic pancreatic cancer animal models (5). The current study demonstrates that 5-FU can prevent tumor cell proliferation and cannabinoids can induce apoptosis of tumor cells. Therefore, using 5-FU in combination of cannabinoids may be synergized antitumor effects and the future research should be conducted.

In conclusion, cannabinoid treatment in mice was not affected by weight gain and blood profiles. It can induce apoptosis and inhibit the proliferation of human pancreatic ductal adenocarcinoma cells in a dose-dependent manner. This study suggested that cannabinoids have an antitumor effect on a human pancreatic ductal adenocarcinoma cell line (Capan-2)-derived xenograft mouse model.

There are limitations to this study that should be noted; only the content of THC and CBD in the cannabis extract was quantified, and the effect of other compounds, e.g., minor cannabinoids, flavonoids, and terpenes might also contribute to the antiproliferative effect and mediation of apoptosis. Cannabis is a complex plant, more than 60 cannabinoid compounds have been reported in the cannabis extract but CBD and THC are the major compounds and have been most researched in both human patients and animal models (50). However, the therapeutic effects of other cannabinoids have been reported in several diseases including cancer (9). Therefore, the antiapoptosis effects in this study may be synergized by other cannabinoids in cannabis extract. Another limitation was that the anti-caspase-3 antibody used in this present study reacts with both proenzyme and active form of caspase-3 so that the immunoreactivity of caspase-3 in this study may not specifically reflect cell apoptosis so immunohistochemical staining specific for cleaved caspase-3 and more techniques such as Western blot analysis or TUNEL assay should be further performed.

## Data Availability Statement

The raw data supporting the conclusions of this article will be made available by the authors, without undue reservation.

## Ethics Statement

The study protocol was approved by the Institutional Animal Care and Use Committee (IACUC) of The National Cancer institute and Lerdsin Hospital, Department of Medical Services.

## Author Contributions

SSak was responsible for the animal experiment, data analysis, data interpretation, drafting of the manuscript, and approval of the submitted manuscript. NM was involved in cell culture preparation, animal model, data analysis, interpretation, and manuscript drafting and revision. SSan, ST, and AS were responsible for the conception of the study and supervised the research. NS, CB, and VK were responsible for the preparation of cannabinoid solutions and supervised the research. KS was involved in laboratory work, result analysis, and drafting of the manuscript. PS involved in supervising the research including laboratory animal study. KR was responsible for the animal experiment, data analysis, data interpretation, and manuscript editing and revision. All authors contributed to the article and approved the submitted version.

## Conflict of Interest

NS, PP, CB, and VK were employed by The Government Pharmaceutical Organization. The remaining authors declare that the research was conducted in the absence of any commercial or financial relationships that could be construed as a potential conflict of interest.

## Publisher's Note

All claims expressed in this article are solely those of the authors and do not necessarily represent those of their affiliated organizations, or those of the publisher, the editors and the reviewers. Any product that may be evaluated in this article, or claim that may be made by its manufacturer, is not guaranteed or endorsed by the publisher.

## Abbreviations

CBD: Cannabidiol
5-FU: 5-fluorouracil
PCNA: Proliferating cell nuclear antigen
THC: Delta-9-tetrahydrocannabinol.
